# RAmpSim: a thermodynamic simulator for hybridization capture in metagenomic sequencing

**DOI:** 10.1093/bioinformatics/btag303

**Published:** 2026-07-07

**Authors:** Aidan Zhang, Christina Boucher, Noelle Noyes, Yun William Yu

**Affiliations:** Ray and Stephanie Lane Computational Biology Department, School of Computer Science, Carnegie Mellon University, Pittsburgh, PA 15213, United States; Department of Computer and Information Sciences and Engineering, University of Florida, Gainesville, FL 32611, United States; Department of Veterinary Population Medicine, College of Veterinary Medicine, University of Minnesota, St. Paul, MN 55108, United States; Ray and Stephanie Lane Computational Biology Department, School of Computer Science, Carnegie Mellon University, Pittsburgh, PA 15213, United States

## Abstract

**Motivation:**

Simulators that generate synthetic datasets help address the lack of ground truth for developing and benchmarking computational tools. Many read simulators assume uniform sampling across reference genomes; however, for newer capture-based sequencing technologies (e.g. TELSeq), this assumption is intentionally broken to oversample regions of interest. Along with systematic biases arising from probe multiplicity, sequence composition, and species abundances inherent to capture-based sequencing, this mismatch between modeling assumptions and the characteristics of real data necessitates the design of a new capture-based sequencing-specific simulator.

**Results:**

We present RAmpSim, a fast simulator that models bait–target hybridization and fragment capture using a thermodynamic nearest-neighbor energy model and Boltzmann-weighted sampling of binding sites. Fragments are generated through multinomial sampling parameterized by bait concentration, binding energy, and genomic abundance before being passed to existing models of platform-specific errors. Implemented in Rust, RAmpSim reproduces empirical within-genome coverage and cross-species enrichment patterns observed in capture-based metagenomic datasets. RAmpSim generally outperforms a uniform baseline with respect to position-based earth mover’s distance when compared against the empirical coverage distribution. Classification analysis also shows high recall in recovering empirical high-coverage regions while outperforming a uniform baseline.

**Availability:**

Code, example scripts, and data sources are available at https://github.com/az002/RAmpSim.git.

## 1 Introduction

Shotgun metagenomics has substantially advanced our understanding of microbial communities and their interactions with hosts in a variety of environments and samples, including soil and water in agricultural environments, clinical specimens from human patients, and wastewater used for population-scale surveillance (Sharpton 2014). One of the ongoing challenges that arises with shotgun metagenomics is that genes and genomic regions of interest are frequently dwarfed by extraneous DNA from other microbial species (such as bacteria) or hosts (such as humans). For example, Noyes *et al*. (2016) demonstrated that antimicrobial resistance genes (ARGs)—which define the resistome of a sample—can comprise less than 1% of the sequence data within a shotgun metagenomic sample. This greatly limits our ability to resolve the diversity and composition of such samples, and in practice often necessitates substantially higher sequencing depth or targeted enrichment (e.g. host depletion or capture-based approaches) to recover low-abundance targets with confidence.

Bait-capture, also known as hybridization capture and target enrichment, is a laboratory method for selectively enriching nucleic acid fragments prior to sequencing. In a typical workflow, a complex DNA or cDNA library is incubated with a pool of oligonucleotide baits that are designed to hybridize to regions of interest; bait-target hybrids are then isolated, washed to remove non-hybridized molecules, and prepared to produce a sequencing library that is enriched for the targets. See Bravo *et al*. (2025) for a more detailed discussion of the laboratory procedure. This approach increases sensitivity for low-abundance genes or genomic regions, such as ARGs as discussed above. By limiting off-target reads, sequencing costs are also greatly reduced, enabling more focused studies. For example, bait-capture has been used to study many types of genomic regions, including specific exomes, bacterial species, and viruses, to name a few. Recently, in target-enriched long-read sequencing (TELSeq), bait-capture is combined with long-read sequencing, which further improves the detection sensitivity of rare genes (Slizovskiy *et al*. 2022).

A central challenge in studying bait-capture and developing robust computational methods for the resulting data is the scarcity of ground truth. True target presence, exact on-target fractions, and bias profiles are almost never known for real samples. Simulation provides a practical remedy to this problem through the synthetic generation of a ground truth that can be used to estimate precision and recall. Hence, by generating synthetic datasets with known compositions, various bait designs and novel methods can be systematically evaluated. This use of synthetic data has been paramount to progress in bioinformatics. Simulated reads have routinely enabled the benchmarking of genome assemblers, structural variant callers, and RNA-seq transcript quantification methods (Zerbino and Birney 2008, Rausch *et al*. 2012, Patro *et al*. 2017). Given this importance, there has been a plethora of work in developing accurate and robust read simulators. ART and pIRS were some of the earliest read simulators to simulate Illumina reads from whole genome sequencing experiments (Huang *et al*. 2012, Hu *et al*. 2012). BEAR was one of the earliest shotgun metagenomics read simulators (Johnson *et al*. 2014). PBSIM3 and NanoSim simulate the read length and error profiles of Pacific Biosciences and Oxford Nanopore long-read sequencing data (Yang *et al*. 2017, Ono *et al*. 2022). In these settings, simulators often assume that reads are sampled uniformly from reference genomes, which serves as a useful baseline approximation for untargeted sequencing protocols, including shotgun metagenomics. CapSim uses alignment-based heuristics to simulate capture-based sequencing for single-species samples but does not model the competitive probe-binding common in metagenomic capture sequencing (Cao *et al*. 2018). Although it is possible to design software that works on enriched reads (Shaw *et al*. 2025), such software is typically benchmarked on simulated datasets of lower complexity; their performance on bait-capture enriched reads has in the past only been able to be assessed qualitatively.

In bait-capture protocols, the express purpose is to induce a non-uniform/targeted sampling of genomic loci, thus breaking the uniform-sampling assumption. Bait-target hybridization, washing, and amplification create predictable but uneven coverage patterns. The coverage of the reads usually peaks where the baits tile the genome (i.e. in the targeted regions) and falls off toward the bait edges (i.e., in the non-targeted regions). Enrichment efficiency can also vary substantially with the GC content, fragment length, and other laboratory conditions (e.g. temperature). In addition, near-homologous sequences can lead to off-target fragments being captured and sequenced. In metagenomic samples, these effects compound with abundant host DNA, wide species abundance ranges, and species-specific bait affinities. This bias creates a substantial mismatch between the assumptions of the existing read simulators and the characteristics of the real data. Consequently, benchmarking tools and algorithms on synthetic reads generated under uniform-coverage assumptions from these simulators may not reflect their performance in targeted capture datasets. Methods such as PBSIM3 and NanoSim are capable of producing non-uniform coverage patterns through empirical sampling, however these data-driven approaches require substantial training data, generalize poorly to other laboratory protocols, and offer limited interpretability (Ono *et al*. 2022, Yang *et al*. 2023).

In this paper, we introduce a read simulator that models the thermodynamic interactions between baits and target fragments. We refer to our method as RAmpSim (Read Amplification Simulator). By explicitly incorporating relative bait concentrations, binding energy, and genomic abundance, RAmpSim generates fragment distributions that seamlessly integrate with existing read simulators to produce realistic bait-capture data. Using publicly available datasets, we demonstrate that our method reproduces realistic coverage patterns within and between species in a mock community sample. The resulting simulator provides a reproducible and interpretable foundation for benchmarking bait-capture metagenomic workflows and optimizing bait designs.

## 2 Methods

### 2.1 Simulation overview

Assume that we have a set of baits with known relative concentrations in solution, a set of reference genomes, and additional hyperparameters determining hybridization energetics and the distribution of fragment lengths. We outline the computational steps for simulating bait-based hybridization capture prior to sequencing through the following stages:

1. *Bait-Target Matching*: Baits are mapped to each reference genome using sequence alignment. This gives a set of possible hybridization sites across reference genomes.
2. *Energy-Based Scoring*: Each hybridization site is scored with its binding free energy (ΔG) estimated using a Thermodynamic Nearest-Neighbors model (TNN) with SantaLucia parameters (SantaLucia 1998). For simplicity, we do not assign context-specific mismatch free-energy penalties. Instead, only contiguous exact-match dimers contribute favorably to the thermodynamic score, and mismatches reduce the score indirectly by interrupting these matched runs.
3. *Fragment Sampling*: Through multinomial sampling steps, capture fragment centers are drawn based on the relative concentration of baits near it, their binding probabilities, and the abundance of the reference. Background fragments are sampled uniformly within genomes with coverage depending on genomic abundance. All fragment lengths are determined following a user-defined log-normal distribution.
4. *Error Modeling*: Sequencing errors are introduced using existing read simulators such as PBSIM3 with platform-specific models designed to replicate empirical error profiles for generating realistic reads (Ono *et al*. 2022).

#### 2.1.1 Hybridization model

Consider a metagenomic sample with reference genomes G and abundances ${\left\{a_{g}\right\}}_{g\in G}$. Let $B\subset \Sigma^{*}$ be the set of oligonucleotide baits over the nucleotide alphabet Σ={A,C,G,T}. For baits p∈B, let $H_{p}$ be the set of candidate hybridization sites (x,g) where *x* is the position-indexed sequence of the site within its corresponding reference genome g∈G.

Given a thermodynamic model E(x,p) that estimates ΔG of hybridization between *x* and *p*, we define the unnormalized binding score:


$$S((x,g),p)=a_{g}\;\text{exp}\;\left(-\frac{E(x,p)}{RT}\right)$$

Where the exponential term is the Boltzmann factor, which is an unnormalized relative probability describing the propensity of bait-target binding at equilibrium. In particular, it is a function of the energy E(x,p) and the temperature of the system *T* during hybridization, where *R* is the universal gas constant relating temperature to energy. The abundance term $a_{g}$ weights the score according to the relative representation of the reference in the sample.

In the TELSeq protocol, PCR amplification is performed prior to hybridization, resulting in high fragment multiplicities relative to bait concentrations. In this setting, bait-bait competition can be ignored and bait hybridization can be viewed as saturated. We model the competition among the binding sites for a fixed bait *p* by normalizing over all candidate sites:


$$I((x,g),p)=\frac{S((x,g),p)}{\sum_{b\in H_{p}}S(b,p)}$$

Here I((x,g),p) represents the expected fraction of baits *p* bound to the site (x,g). This is analogous to the formula for fractional occupancy under the Langmuir isotherm model, which is commonly used in DNA microarray hybridization models (Langmuir 1918, Halperin *et al*. 2006). However, in this case, the roles of baits and target fragments are reversed: fragments are viewed as fixed, whereas baits are free to bind to sites distributed among the fragments.

#### 2.1.2 Fragment sampling

We can model the generation of fragments by drawing from a series of multinomial distributions. Since we assume that bait-bait competition is negligible, we model the overall binding rate of each bait *p* as proportional to its relative concentration $c_{p}$ with a shared rate constant. A Poisson model fitted on these rates is used to model the counts of binding events for each bait.

Treating a bait binding event as the capture of a fragment, if we condition on the number of fragments *N*, then the overall process is equivalent to sampling from a multinomial


$$\text{Mult}(N,\pi_{\mathrm{bait}}),\;\pi_{\mathrm{bait}}=\left(\frac{c_{p}}{\sum_{p^{\prime }\in B}c_{p^{\prime }}}:p\in B\right)$$

Similarly, for a bait *p*, we model the rate at which it binds to candidate sites $b\in H_{p}$ as proportional to the score S(b,p), which parameterizes a Poisson distribution. Thus, after conditioning on the number of binding events with bait *p* as $N_{p}=Nw_{p}$, we obtain another multinomial


$$\text{Mult}(N_{p},\pi_{p}),\;\pi_{p}=(I(b,p):b\in H_{p})$$

After drawing the binding site (x,g), we draw a fragment length under an empirically determined user-defined read length distribution, $L∼\text{Lognormal}(\mu ,\sigma^{2})$. This determines a uniform distribution of fragment centers in a window of length *L* centered on the binding site from which the actual fragment is ultimately drawn.

To sample background fragments for a fixed number of fragments $N^{\prime }$, we sample $a_{g}N^{\prime }$ fragments for each genome g∈G uniformly at random across all positions in the genome.

### 2.2 Implementation details

RAmpSim is implemented in Rust for computational efficiency. All computations are performed on a single CPU thread. Reference genomes are loaded into memory for fast access during bait–target evaluation and fragment sampling.

As the primary input, RAmpSim accepts a SAM file containing candidate bait–target alignments generated from read mappers such as Bowtie2 (Langmead and Salzberg 2012). Our mapping criteria excludes indels and allows for at most 40 mismatches, which is in concordance with the binding tolerance described by Noyes *et al*. (2017). From these alignments, fragments are sampled according to the hybridization and fragment-length models. RAmpSim has parameters that determine the fraction of capture compared to the background fragments, the genomic abundances of the present species, the hybridization temperature (used in the Boltzmann-weighted energy model) and the log-normal fragment-length distribution lnμ and lnσ. These parameters can be given by the user or estimated from sequencing libraries.

Both capture-derived and background fragments are output as a FASTA file. On TELSeq-scale datasets (∼10 000 baits producing ∼100000 fragments), a complete simulation before errors are introduced runs in a few seconds on a workstation equipped with an Intel Xeon Gold 5416S CPU and 512 GB of RAM; however, in our simulations of microbial genomes, the peak memory usage was less than 100 MB.

Code and example scripts are available at https://github.com/az002/RAmpSim.git.

### 2.3 Simulation setup (hyperparameter tuning)

To validate RAmpSim, we used publicly available TELSeq datasets (https://www.ncbi.nlm.nih.gov/bioproject/PRJNA751055) sequenced from a vendor-supplied mock microbial community (ZymoBIOMICS Microbial Community DNA Standard II [Log Distribution]) including a mixture of bacterial and fungal species that decrease in genomic abundance by a factor of 10 (Table 1) (Slizovskiy *et al*. 2022). The theoretical abundances are used to weight the bait binding scores described in the hybridization model and determine the relative proportions of background fragments generated from each species.

**Table 1:** Composition of ZymoBIOMICS Microbial Community Standard II.

Organism	Genomic Abundance (%)
*Listeria monocytogenes*	89.100000
*Pseudomonas aeruginosa*	8.900000
*Bacillus subtilis*	0.890000
*Saccharomyces cerevisiae*	0.890000
*Escherichia coli*	0.089000
*Salmonella enterica*	0.089000
*Lactobacillus fermentum*	0.008900
*Enterococcus faecalis*	0.000890
*Cryptococcus neoformans*	0.000890
*Staphylococcus aureus*	0.000089

The genomic abundances for each species are used as the weighting term $a_{g}$ for the binding scores in the hybridization model. The abundances also directly determine the relative proportions of sampled background fragments.

The TELSeq dataset includes reads from three mock community samples, each independently physically fragmented to achieve different target median fragment length distributions (2 kb, 5 kb, and 8 kb) prior to sequencing. To focus on modeling fragment capture, we remove the effects of PCR bias by deduplicating reads using samtools markdup (Li *et al*. 2009). To fit the distribution of fragment lengths, the SciPy stats package was used to determine the μ and σ parameters of a log-normal distribution for each dataset (Virtanen *et al*. 2020). To estimate the proportion of capture-derived and background fragments, reads within μ/2 of candidate bait binding sites were considered capture reads and the rest as background, according to the length *L* window used to generate fragments in RAmpSim. The temperature for each dataset was set to 70 ${}^{{}^{\circ}}$C based on the protocol outlined by Slizovskiy *et al*. (2022). Histograms of fragment length distributions with fitted log-normal curves and a table of capture to background fragment ratios with other model hyperparameters are shown in Supplementary Fig. 1 and Supplementary Table 1, available as supplementary data at *Bioinformatics* online respectively.

The generated fragments are used as input to PBSIM3 using the template strategy and ERRHMM-Sequel model. We simulate reads with one pass per molecule, which yields a higher error rate than the ≈5-pass CCS reads in the TELSeq datasets. Our evaluation focuses on metrics based on fragment locations and multiplicities such as coverage EMD, peak recall, and genomic abundance. We do not expect this discrepancy in error rate to materially affect our conclusions as these metrics are robust against per-base accuracy.

## 3 Results

### 3.1 Fidelity of coverage patterns

We compute normalized coverage distributions for both simulated and experimental data by dividing the coverage at each position within a genome by the total coverage across all positions. The simulated coverage profiles from our method closely reproduce the empirical normalized coverage patterns for large contiguous genomes across all replicates at log scale. For both simulated and experimental data, coverage levels are highest near regions with high bait density and decay to background levels otherwise (Fig. 2). Coverage distributions for additional genomes and replicates are shown in Supplementary Figs. 2 and 3, available as supplementary data at *Bioinformatics* online.

**Figure 1:**
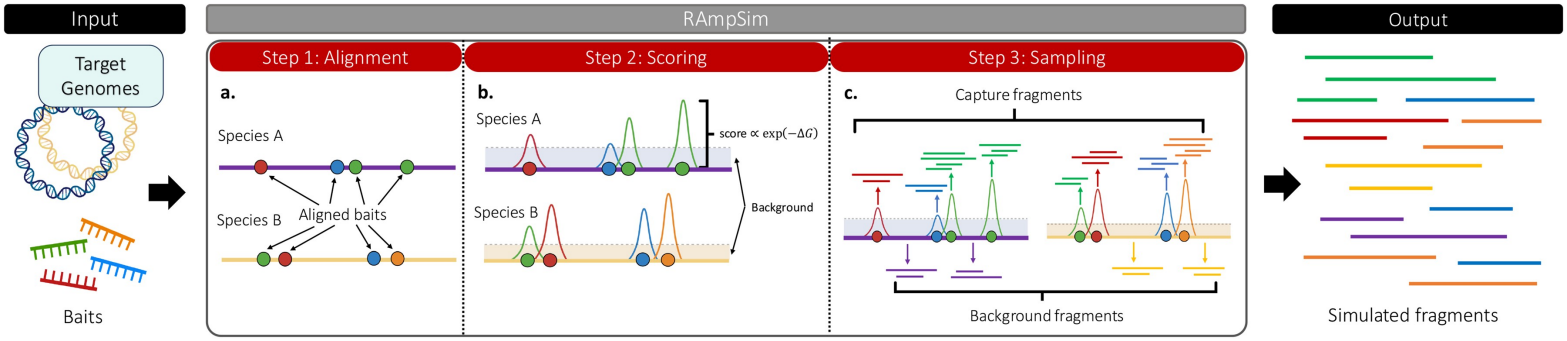
Simulation framework for generating fragments. (a) Bait sequences are aligned to each reference genome present within the sample, allowing secondary mappings to capture off-target hybridization sites. (b) Candidate binding sites discovered from the alignment step are scored using their hybridization free energies (ΔG). Energies across candidate sites for a given bait are normalized with the sum of their abundance-weighted scores. (c) Capture fragments are sampled according to multinomial distributions on baits and their binding sites while background fragments are sampled uniformly across positions.

**Figure 2:**
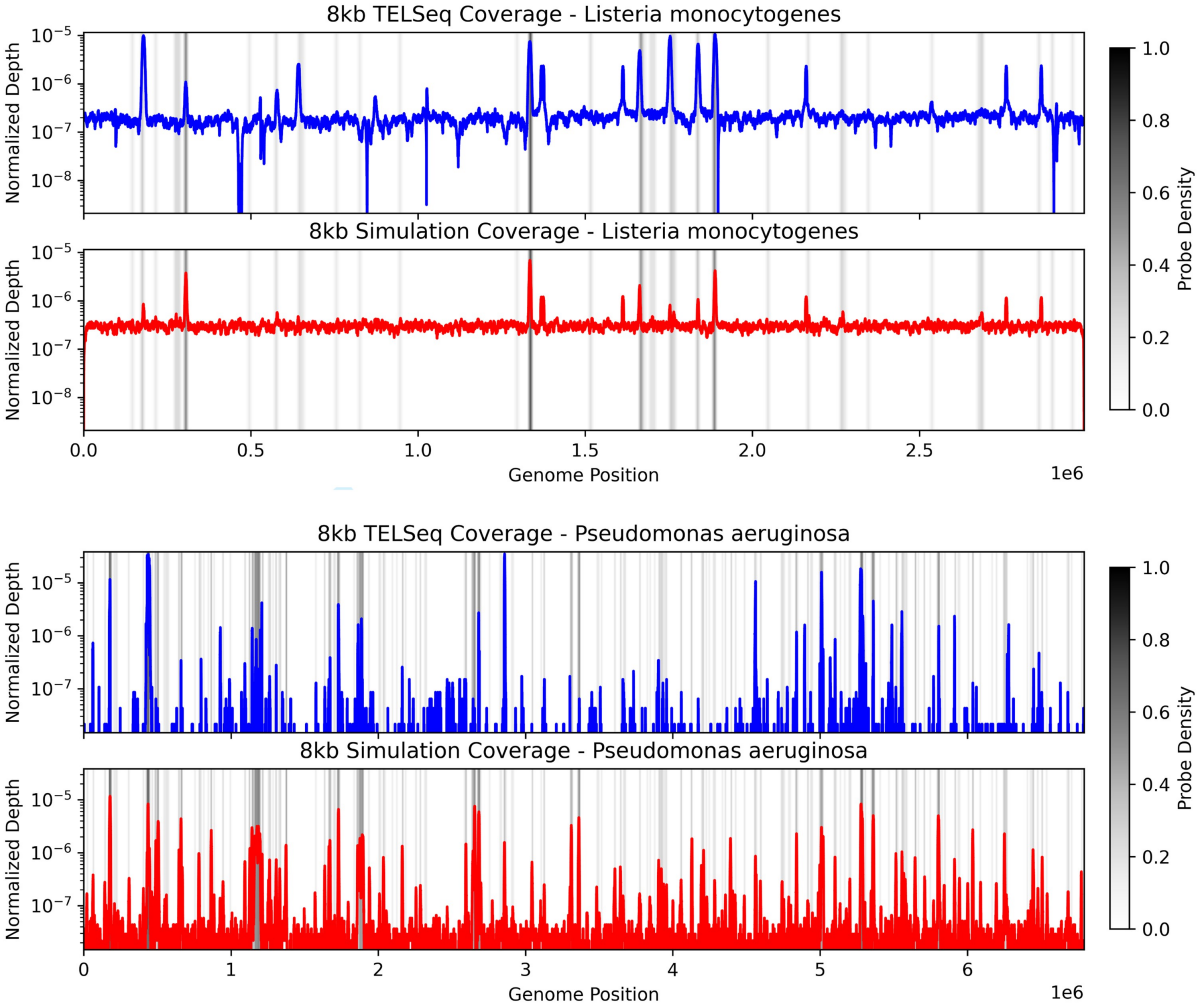
Log coverage distribution plots for *L. monocytogenes* (top) and *P. aeruginosa* (bottom). The coverage distribution for each genome was normalized by the sum of the coverage over each position. The coverage patterns between the observed (blue) and simulated (red) data are similar, in which peaks in each distribution correspond to high bait-density regions shown in gray. Outside of these regions, coverage falls to background levels. Coverage distributions for additional genomes and replicates are shown in Supplementary Figs. 2 and 3, available as supplementary data at *Bioinformatics* online.

We quantify similarity between the observed and simulated coverage distributions using a position-based earth mover’s distance (EMD), which can be interpreted as the average genomic distance that normalized coverage mass must be moved to transform one distribution into the other after coordinates are scaled to[0,1]. Lower EMD values indicate closer agreement in the locations and relative weights of enriched regions. For species with multiple references, we report length-weighted averages of per-reference EMD values so that each species-level summary reflects whole-genome agreement. Compared to a uniform baseline, RAmpSim generally yields lower EMD with respect to the empirical coverage distributions. The main exceptions are *S. aureus*, which is worse than the uniform baseline in all three replicates, and *L. fermentum* in the 5 kb replicate. The *L. fermentum* result is likely related to the absence of baited target regions in its genome, making its empirical coverage profile comparatively background-dominated, whereas the *S. aureus* exception is mainly due to the simulator producing additional enriched regions absent in the observed coverage distribution (Supplementary Fig. 4, available as supplementary data at *Bioinformatics* online). EMD results for additional replicates are provided in Supplementary Table 2, available as supplementary data at *Bioinformatics* online.

To evaluate the simulator’s ability to recover high-coverage regions, we frame the problem as a binary classification task. Positions within each genome are classified as high coverage based on whether their raw coverage exceeds the genome-wide median. We apply this procedure to both the observed and simulated data, treating the observed high-coverage positions as ground truth. For most genomes, the F1 score is at least 0.5 with the recall being consistently higher than the precision (Table 3). The high recall indicates that RAmpSim reproduces most enriched coverage regions found in the observed data, whereas the lower precision indicates that background or off-target positions often pass the median threshold. In particular, RAmpSim tends to produce additional off-target high-coverage regions. This effect is strongest in weak-signal genomes, where sparse empirical enrichment makes precision especially sensitive to extra simulated peaks. Thus, *S. aureus* can show poorer EMD than the uniform baseline despite exhibiting substantially better peak recovery, because extra unsupported peaks increase the transport cost by forcing normalized coverage mass away from simulated loci that are absent empirically.

**Table 2:** Position-based, length-weighted EMD results for the 8 kb replicate.

Species	EMD(tels, sims)	EMD(tels, unif)
*Listeria monocytogenes*	**0.037**	0.047
*Pseudomonas aeruginosa*	**0.074**	0.149
*Bacillus subtilis*	**0.036**	0.045
*Escherichia coli*	**0.036**	0.038
*Enterococcus faecalis*	**0.222**	0.299
*Lactobacillus fermentum*	**0.201**	0.349
*Salmonella enterica*	**0.047**	0.117
*Staphylococcus aureus*	0.259	**0.200**

Lower values indicate closer agreement between the empirical and simulated positional coverage distributions (best values bolded). For species with multiple references (e.g. chromosome and plasmids), values are length-weighted averages of per-reference EMDs. Eukaryotic species such as *S. cerevisiae* and *C. neoformans* were not considered targets in the original bait design. Tables for additional replicates are shown in Supplementary Table, available as supplementary data at *Bioinformatics* online LABE50: tab: emd_2_5kb.

**Table 3:** F1, recall, and precision scores of high/low coverage classification by RAmpSim and a uniform baseline in the 8 kb replicate.

Species	RAmpSim			Uniform baseline		
	F1 Score	Recall	Precision	F1 Score	Recall	Precision
*Listeria monocytogenes*	**0.5030**	**0.5119**	0.4944	0.477±0.024	0.480±0.030	0.474±0.024
*Pseudomonas aeruginosa*	**0.3152**	**0.5312**	**0.2240**	0.233±0.019	0.379±0.031	0.169±0.014
*Bacillus subtilis*	**0.3678**	**0.5524**	**0.2756**	0.287±0.027	0.487±0.052	0.204±0.019
*Escherichia coli*	**0.7661**	**0.7879**	**0.7455**	0.374±0.027	0.402±0.030	0.350±0.024
*Salmonella enterica*	**0.7202**	**0.8518**	**0.6239**	0.347±0.023	0.480±0.032	0.271±0.018
*Lactobacillus fermentum*	**0.0351**	**0.4105**	**0.0183**	0.000±0.000	0.000±0.000	0.000±0.000
*Enterococcus faecalis*	**0.4995**	**0.9813**	**0.3350**	0.058±0.041	0.189±0.133	0.034±0.024
*Staphylococcus aureus*	**0.1743**	**0.9878**	**0.0956**	0.033±0.053	0.034±0.055	0.033±0.048

Uniform baseline values are reported as mean ±95% confidence intervals over N=100 simulated uniform-coverage datasets (best values bolded). For each species, the left block reports the scores obtained by RAmpSim, and the right block reports the corresponding uniform-baseline metrics. For all species except *L. monocytogenes*, the F1, recall, and precision for the high-coverage regions called by RAmpSim lie above the 95% confidence intervals of the uniform baseline. See Supplementary Table 2, available as supplementary data at *Bioinformatics* online for corresponding F1 metrics on the 2 kb and 5 kb replicates.

We also compare the F1, recall, and precision against a random uniform baseline. For N=100 iterations, random uniform distributions were generated by sampling fragment start positions uniformly across each genome, with fragment lengths drawn from the fitted log-normal distributions. The number of fragments was chosen so that the expected mean coverage matched the empirical average coverage depth. We then applied the same coverage-classification pipeline to these simulated coverages and summarized the resulting metrics as mean±95% confidence intervals. For most species, the F1, recall, and precision achieved by RAmpSim are higher than the corresponding uniform-baseline intervals, whereas performance for *L. monocytogenes* is only modestly better than the baseline, consistent with its coverage distribution being close to uniform. Additional F1 metrics for the 2 kb and 5 kb replicates are reported in Supplementary Table 3, available as supplementary data at *Bioinformatics* online. A complementary strict exact probe-footprint analysis of intended versus unintended capture is provided in Supplementary Table 4, available as supplementary data at *Bioinformatics* online, where RAmpSim shows substantially closer agreement with the empirical off-target burden than the uniform baseline under this conservative definition. For example, in the 8 kb strict probe-footprint analysis, empirical off-target burden was 0.108, compared with 0.151 for RAmpSim and 0.978 for the uniform baseline.

**Figure 3:**
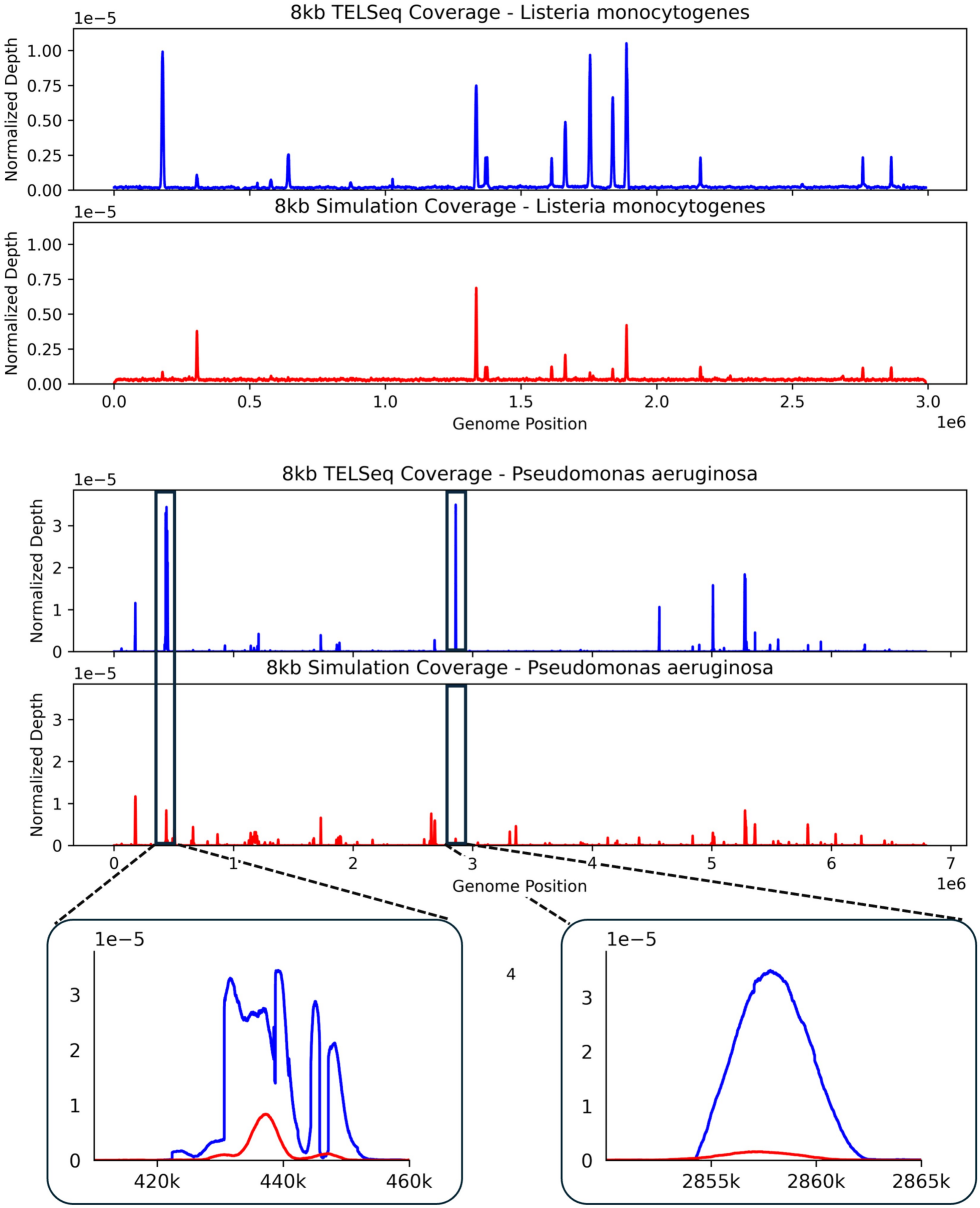
Normalized coverage distributions for *L. monocytogenes* (top) and *P. aeruginosa* (bottom). Although the simulator approximates the locations of peaks, the relative heights of peaks can differ substantially between the simulated and experimental data. We show two locations in the *P. aeruginosa* genome (∼440 kb and ∼2.86 Mb) where the difference is largest. Coverage distributions for additional genomes are shown in Supplementary Figs. 2 and 3, available as supplementary data at *Bioinformatics* online.

To make differences between the simulated and observed coverage distributions more apparent, we plot the coverage profiles on a common scale, with discrepancies especially pronounced for *P. aeruginosa*. In particular, prominent coverage peaks at approximately 0.5 and 3 Mb in the experimental data are markedly attenuated in the simulated data (Fig. 3). This may be partially explained by the prominence of the background distribution pulling away density from peaks in the simulated coverage profile.

We further benchmark the accuracy of our thermodynamic binding model against CapSim, an established heuristic simulator for target capture. Since CapSim is designed for single-species inputs, we generated a synthetic reference by concatenating all mock community genomes into a single input file, effectively simulating a community with uniform abundance. Under this condition, the normalized within-species read coverage distributions generated by RAmpSim align with those from CapSim apart from a lack of background reads from CapSim (Supplementary Fig. 5, available as supplementary data at *Bioinformatics* online). This confirms that our thermodynamic model correctly reproduces established sequence-dependent capture biases identified by standard alignment-based heuristics. However, assuming uniform abundance in mixed biological samples does not reflect real biological data, so this limits the general applicability of CapSim.

### 3.2 Fidelity of species abundances

In addition to within-species coverage patterns, we evaluate RAmpSim’s ability to reproduce the abundances found in observed data  relative to the theoretical genomic abundances. Abundances were computed by assigning reads to species based on unique QC-filtered minimap2 alignments outlined by Nicholls *et al*. (2018).

In general, the simulated and observed data show similar abundance patterns (Table 4). For example, in both datasets, most reads map to *L. monocytogenes*, which has the highest theoretical genomic abundance in the mock sample. Furthermore, target species such as *E. coli*, *S. enterica*, and *B. subtilis* in both the simulated and observed data show substantially increased abundance compared to the theoretical compositions as the bait design for the community selectively targets ARGs present in each genome; non-target species (due to their lack of ARGs) such as *S. cerevisiae*, *C. neoformans*, and *L. fermentum* either showed suppressed abundance or remained having the lowest abundance relative to other species. Additional tables for the remaining replicates are shown in Supplementary Table 5, available as supplementary data at *Bioinformatics* online.

**Table 4:** Comparison of theoretical, observed, and simulated abundances of the 8 kb replicate.

Species	Theoretical	Observed	Simulated
*Listeria monocytogenes*	89.100000	64.893325	35.984226
*Pseudomonas aeruginosa*	8.900000	9.759034	26.771994
*Bacillus subtilis*	0.890000	9.183611	2.717070
*Saccharomyces cerevisiae*	0.890000	0.090746	0.370099
*Escherichia coli*	0.089000	10.069688	14.768590
*Salmonella enterica*	0.089000	5.964689	15.031289
*Lactobacillus fermentum*	0.008900	0.000993	0.189728
*Enterococcus faecalis*	0.000890	0.026362	1.739722
*Cryptococcus neoformans*	0.000890	0.000000	0.000000
*Staphylococcus aureus*	0.000089	0.010560	2.237555

In general the changes in genomic abundances compared to the theoretical values are preserved between the observed and simulated data. *L. monocytogenes* remains the most abundant species, and species targeted by the bait design such as *B. subtilis*, *E. coli*, and *S. enterica* are enriched; non-target species, including *L. fermentum*, *C. neoformans*, and *S. cerevisiae* remain depleted or remain having the lowest abundance. The main discrepancies are the absolute changes between the simulated and observed abundances, being most prominent for *L. monocytogenes* and *P. aeruginosa*. See Supplementary Table 5, available as supplementary data at *Bioinformatics* online for the abundance comparisons of remaining replicates.

Although the overall abundance patterns between the simulated and observed data are similar, there are nontrivial differences in the absolute abundance values of species between the datasets. For example, the abundance of *L. monocytogenes* is much lower in the simulated data compared to the observed data, whereas species such as *P. aeruginosa* and *S. enterica* are even more enriched in the simulated data. In particular, *P. aeruginosa* is known to have high GC content leading to inefficiencies in DNA extraction, which could explain the substantial increase in abundance in the simulated data compared to the observed data.

To broaden the empirical scope beyond the main benchmark, we also applied RAmpSim to a human gut microbiome mock dataset (ZymoBIOMICS Gut Microbiome Standard). We used the same general workflow as above with fitted fragment-length distributions from observed data without further extensive dataset-specific tuning. Across 21 computable taxa, agreement was variable (median EMD =0.132, median F1 =0.145). Several *E. coli* references showed the strongest agreement, whereas other taxa showed weaker agreement. Representative empirical-versus-simulated coverage profiles and metrics are provided in Supplementary Fig. 6 and Supplementary Table 6, available as supplementary data at *Bioinformatics* online with abundance comparisons in Supplementary Table 7, available as supplementary data at *Bioinformatics* online. We therefore treat this mock gut microbiome analysis as an additional supplementary transferability check rather than a second optimized benchmark.

## 4 Discussion

The results show that RAmpSim is able to recover large-scale coverage structures observed in bait-capture sequencing experiments. For the majority of bacterial genomes with a single main chromosome, simulated coverage peaks coincide with high bait-density regions and decay to background elsewhere, matching the empirical profiles reported in Fig. 2 and Supplementary Figs. 2 and 3, available as supplementary data at *Bioinformatics* online. Additionally, validation against the heuristic simulator CapSim confirmed that RAmpSim reproduces expected normalized within-species coverage profiles. Together with the low earth mover’s distances between simulated and observed coverage distributions (Table 2; Supplementary Table 2, available as supplementary data at *Bioinformatics* online), this suggests that a model that combines alignment-derived candidate hybridization sites, thermodynamics-based hybridization scores, and abundance-weighted sampling is sufficient to explain the dominant enrichment patterns in this dataset.

One of the main remaining discrepancies between simulated and experimental data concerns the exact intensities of coverage peaks rather than the broad placement of enriched regions. These discrepancies arise in two settings: (1) peaks present in the experimental data but absent or attenuated in the simulated data, and (2) differences in the relative heights of peaks within the same genome (Fig. 3; Supplementary Figs. 2 and 3, available as supplementary data at *Bioinformatics* online). Both effects reflect a smoothing of coverage distributions generated from RAmpSim relative to observed data: strong TELSeq peaks often appear attenuated in RAmpSim, while sparse inter-peak regions retain more coverage in the simulation. These discrepancies likely reflect intentional simplifications in the current model, including the absence of explicit GC-dependent effects on hybridization and amplification, which may lead to over-enrichment of some GC-rich regions; a simplified binding model that can overstabilize near-matching off-target sites; a broader background component that reduces peak-to-background contrast; and an equilibrium approximation that may underestimate the sharper contrast produced by non-equilibrium hybridization and wash steps.

Coverage classification analysis is consistent with this interpretation. Recall is high across most genomes, indicating that RAmpSim reproduces most empirical high-coverage locations (Table 3; Supplementary Table 3, available as supplementary data at *Bioinformatics* online). Precision is generally lower, indicating that the simulator also predicts additional enriched loci that are not strongly supported in the empirical data. This effect is especially apparent for *S. aureus* and *L. fermentum*. Specifically, this edge case appears to reflect weak empirical enrichment more than abundance alone: low-abundance species are often affected, but weakly targeted or near-uniform genomes can show similar behavior when observed coverage remains close to background. In these low-signal settings, small amounts of excess simulated enrichment disproportionately reduce precision and increase position-based EMD. Thus, the dominant failure mode of RAmpSim is not systematic misplacement of enrichment, but over-enrichment of some unsupported loci together with imperfect calibration of relative peak intensities.

To assess whether this performance could be reproduced by a standard uniform model commonly used by non-capture read simulators, we compared F1, recall, and precision to a uniform baseline in which fragments were sampled uniformly along each genome, with lengths drawn from the fitted fragment-length distributions and the number of fragments chosen to match the empirical mean coverage. Across most bacterial species, the F1, recall, and precision of RAmpSim lie above the corresponding 95% empirical intervals of the uniform baseline (Table 3; Supplementary Table 3, available as supplementary data at *Bioinformatics* online), indicating that the observed agreement with TELSeq high-coverage positions cannot be explained by uniform fragmentation alone. For low-abundance species such as *E. faecalis* and *S. aureus*, the uniform baseline yields very low F1 scores, whereas RAmpSim still recovers most observed high-coverage locations, albeit with additional off-target enriched loci. In contrast, performance for *L. monocytogenes* is only modestly better than the uniform baseline, consistent with its observed coverage distribution being close to uniform.

At the species level, the simulator also recapitulates the qualitative composition of the TELSeq sample. The dominant species in the mock community remains dominant, targeted bacterial species are enriched relative to their theoretical proportions, and non-target fungal and bacterial species remain depleted (Table 4; Supplementary Table 5, available as supplementary data at *Bioinformatics* online). This highlights a key advantage of RAmpSim over single-species simulators such as CapSim, which do not natively model probe competition across a community. The remaining quantitative discrepancies, including underrepresentation of *L. monocytogenes* and overrepresentation of *P. aeruginosa* and *S. enterica*, are consistent with biases not included in the current model, particularly GC-dependent inefficiencies during extraction, library preparation, and capture. In addition, the simplified thermodynamic model may still assign overly favorable scores to some near-matching off-target interactions, which could further distort species-level abundances.

Taken together, these observations point to several extensions. First, incorporating an explicit GC-dependent weighting term, either at the hybridization stage or at a separate amplification stage, should improve the fit for high-GC genomes and reduce peak flattening. Second, replacing the simplified TNN model with a more complete parameterization of sequence-dependent free energies would reduce systematic differences in species-level abundances. These extensions can be added without changing the overall structure of the simulator and while retaining its current runtime characteristics. In practice, this suggests that RAmpSim is already well suited for reproducing coarse enrichment structure and qualitative abundance patterns and for benchmarking downstream analyses that depend primarily on relative enrichment.

## 5 Conclusion

In summary, RAmpSim reproduces the major spatial and compositional features of the TELSeq dataset. Its single-threaded, memory-resident implementation makes it a practical tool for evaluating bait designs, for stress-testing downstream analysis pipelines under controlled conditions, and for studying how individual sources of bias (GC content, bait density, species abundance) propagate to observable coverage. As richer empirical datasets become available, the simulator can be iteratively calibrated using the same framework described here.

## Supplementary Material

btag303_Supplementary_Data

## Data Availability

TELSeq sequencing data is available from NCBI BioProject PRJNA751055. Other data is available from the associated Github repo at https://github.com/az002/RAmpSim
