# Supplementary material for: RAmpSim: a thermodynamic simulator for hybridization capture in metagenomic sequencing
Source: Bioinformatics. 2026 Jul 7;42(Suppl 1):btag303. doi: 10.1093/bioinformatics/btag303 (PMC13341137; doi:10.1093/bioinformatics/btag303)
Supplement: btag303_Supplementary_Data [file btag303_supplementary_data.pdf]

Supplementary Material

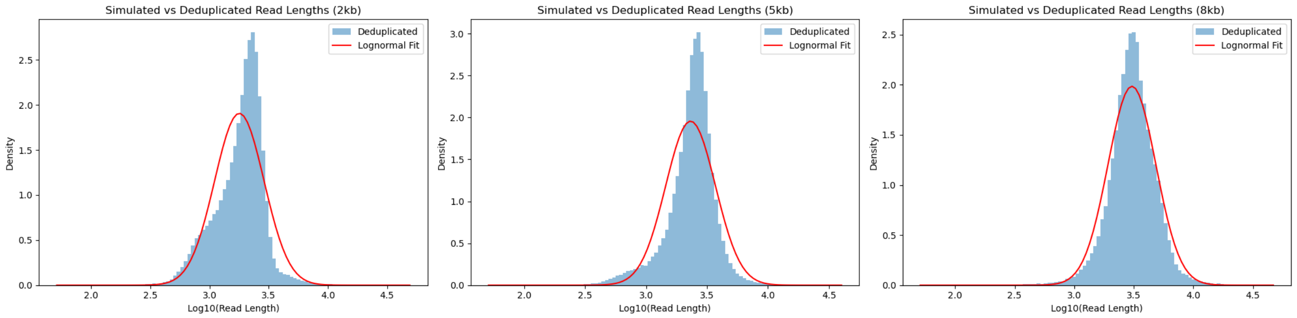

Fig. S1. Fitted log-normal distributions of fragment lengths across replicates. We see that on the log-scale each distribution is approximately normal, and that the model fit improves for larger target fragment lengths. In particular, the model fit is best near the median.

Table S1. Simulation Parameters by Insert Size

Insert Size	–nfrag	–flen	–lognorm-sd	–split
8kb	154000	3000	0.46	0.63
5kb	111000	2300	0.47	0.68
2kb	125000	1800	0.48	0.85

Species	2kb		5kb	
	EMD(tels, RAmpSim)	EMD(tels, unif)	EMD(tels, RAmpSim)	EMD(tels, unif)
Listeria monocytogenes	**0.067**	0.095	**0.037**	0.050
Pseudomonas aeruginosa	**0.057**	0.133	**0.084**	0.154
Bacillus subtilis	**0.014**	0.053	**0.042**	0.044
Escherichia coli	**0.041**	0.043	**0.032**	0.035
Enterococcus faecalis	**0.232**	0.257	**0.196**	0.278
Lactobacillus fermentum	**0.168**	0.297	0.278	**0.133**
Salmonella enterica	**0.050**	0.127	**0.051**	0.129
Staphylococcus aureus	0.232	**0.179**	0.172	**0.139**

Table S2. Position-based, length-weighted EMD results for the 2kb and 5kb replicates. Lower values indicate closer agreement between the empirical and simulated positional coverage distributions. Eukaryotic species such as *S. cerevisiae* and *C. neoformans* contain multiple chromosomes of varying lengths and were not considered targets in the original bait design.

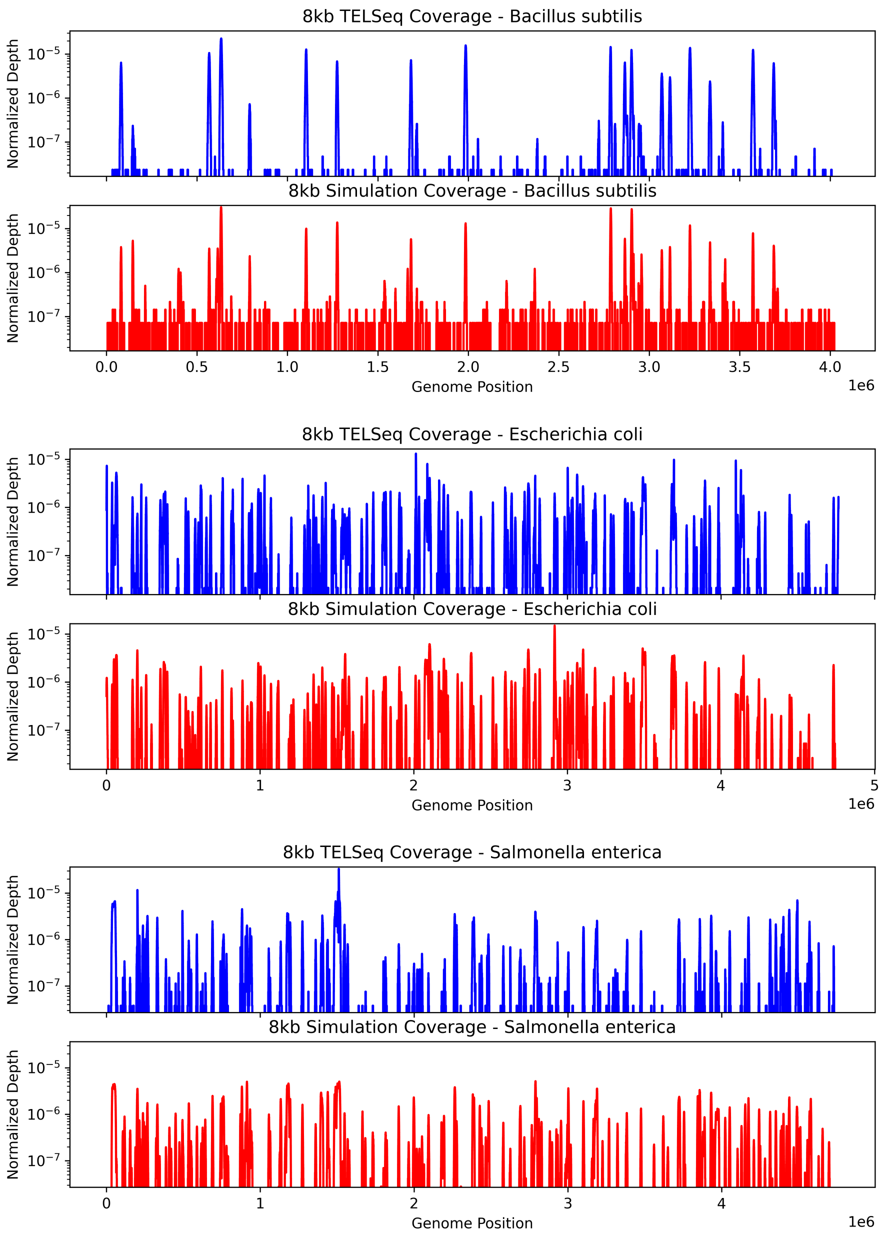

Fig. S2. Log coverage distribution plots for *B. subtilis* (top), *E. coli* (middle), and *S. enterica* (bottom). The coverage distribution for each genome was normalized by the sum of the coverage over each position. The coverage patterns between the observed (blue) and simulated (red) data are similar, in which peaks in each distribution correspond to high bait-density regions shown in gray. Outside of these regions, coverage falls to background levels.

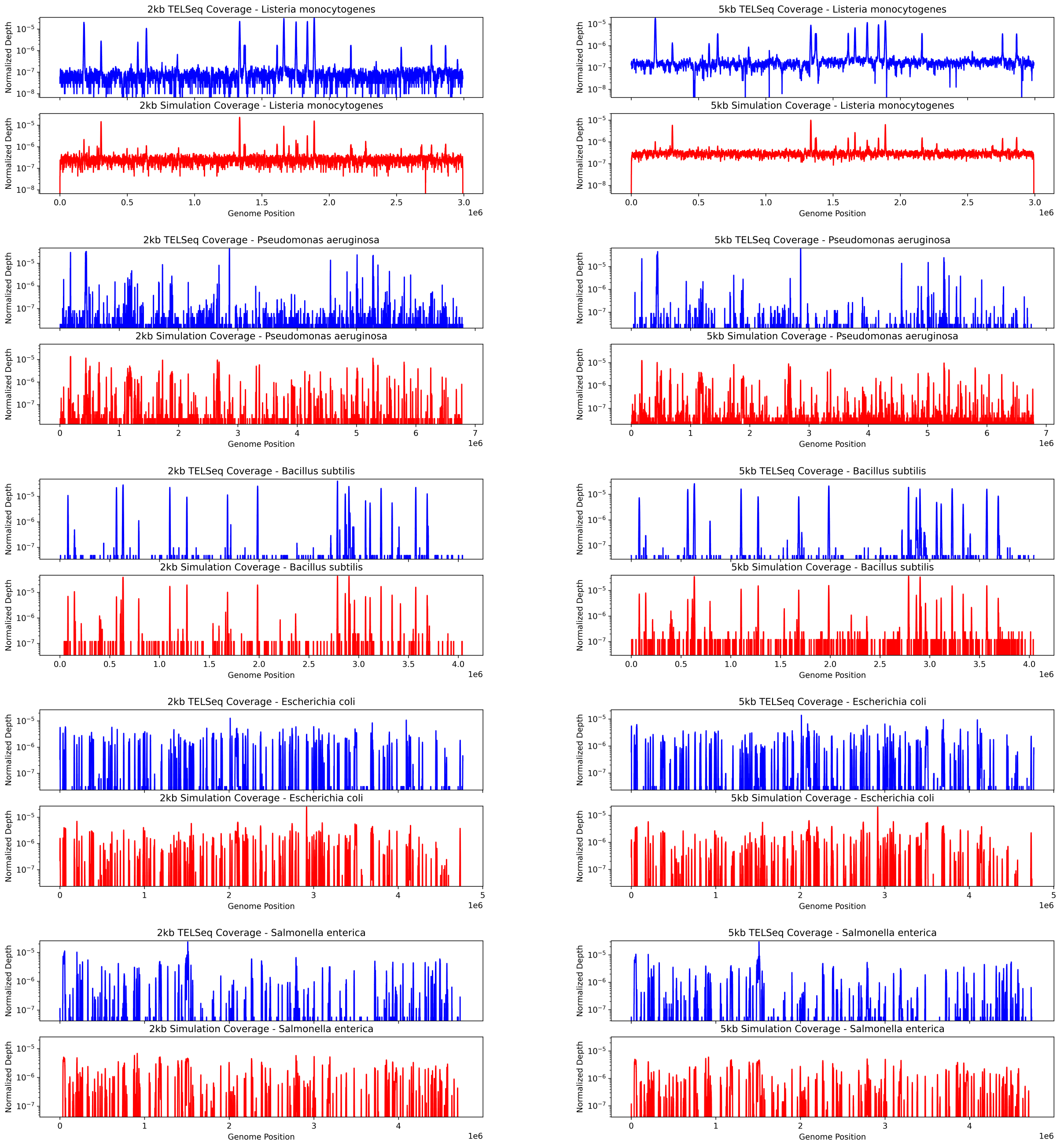

Fig. S3. Log coverage distribution plots of large genomes for 2kb and 5kb replicates. The coverage distribution for each genome was normalized by the sum of the coverage over each position. Similar to the coverage patterns in the 8kb replicates, the observed (blue) and simulated (red) data for the 2kb and 5kb replicates share similar coverage patterns.

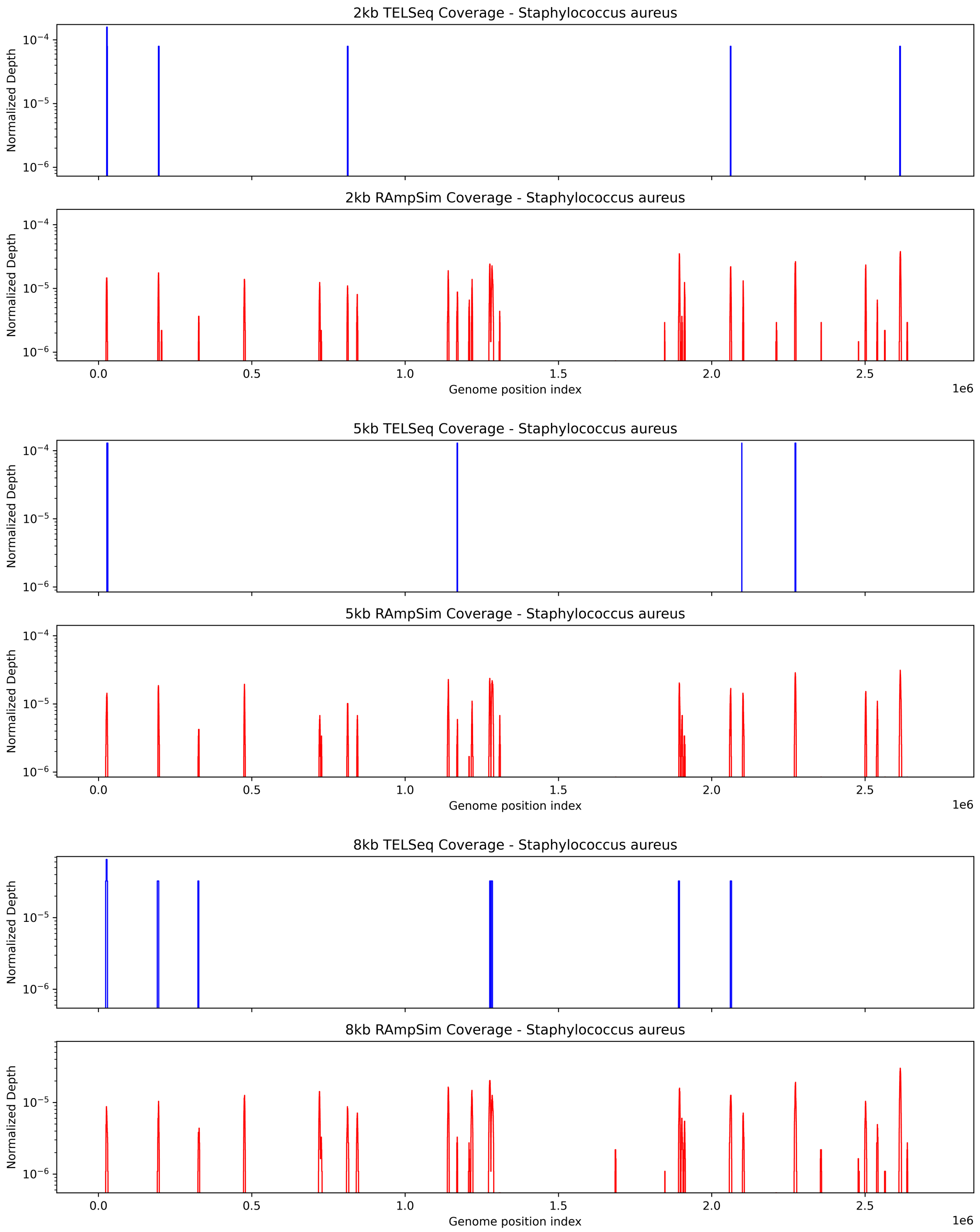

Fig. S4. Log coverage distribution plots of *S. aureus* for all replicates. The coverage distribution for each genome was normalized by the sum of the coverage over each position. For each replicate RAmpSim produces additional enriched regions not present in the observed coverage distribution.

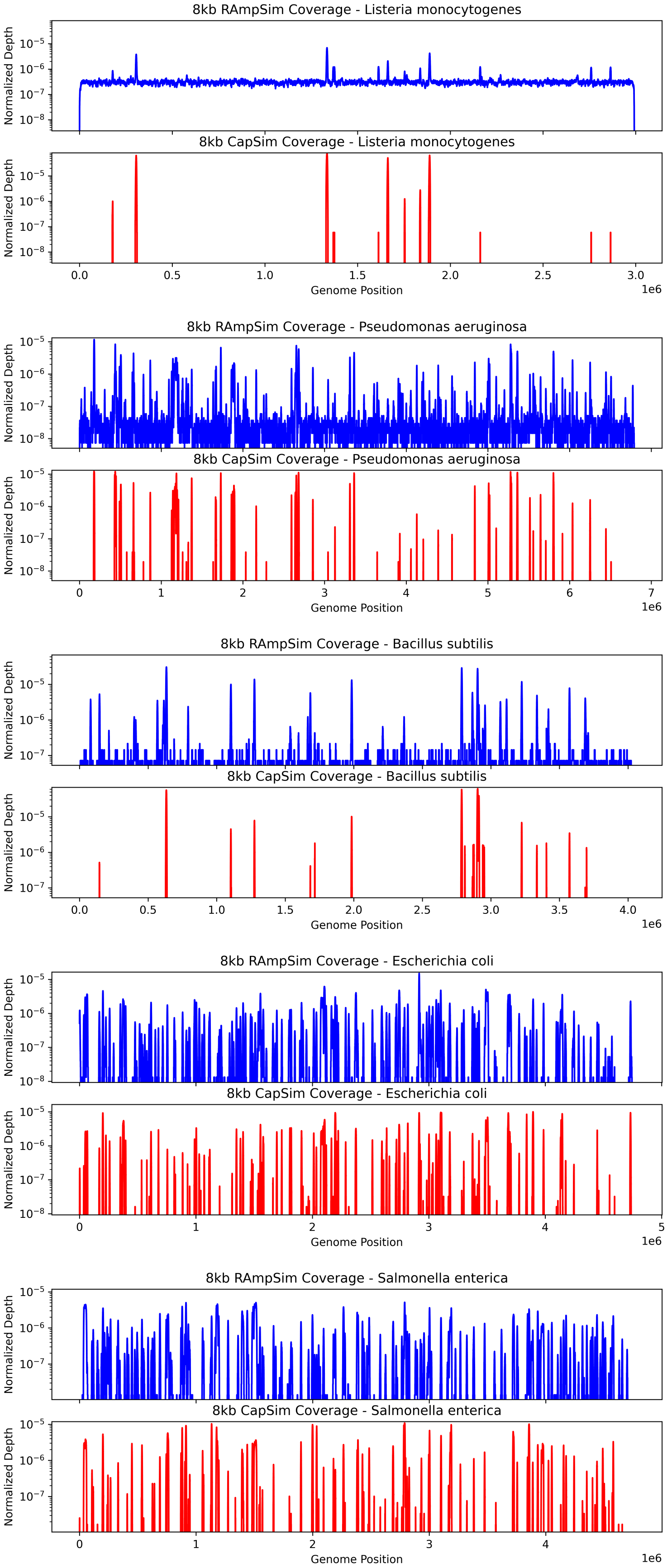

Fig. S5. Log coverage distribution plots of large genomes for 8kb replicates from reads generated by RAmpSim (blue) and CapSim (red). The coverage distribution for each genome was normalized by the sum of the coverage over each position. Both simulators generally agree on the location of peaks, but CapSim does not produce background reads.

Species	RAmpSim (2kb)			Uniform baseline (2kb)		
	F1 Score	Recall	Precision	F1 Score	Recall	Precision
Listeria monocytogenes	**0.4864**	**0.5023**	**0.4714**	0.453 ± 0.022	0.461 ± 0.028	0.446 ± 0.019
Pseudomonas aeruginosa	0.3269	0.3156	**0.3391**	0.318 ± 0.014	0.447 ± 0.022	0.247 ± 0.011
Bacillus subtilis	**0.3570**	0.3663	**0.3480**	0.196 ± 0.024	0.405 ± 0.052	0.129 ± 0.015
Escherichia coli	**0.7785**	**0.8296**	**0.7333**	0.289 ± 0.035	0.409 ± 0.095	0.225 ± 0.018
Salmonella enterica	**0.7286**	**0.8710**	**0.6263**	0.245 ± 0.034	0.363 ± 0.115	0.187 ± 0.016
Lactobacillus fermentum	0.0000	0.0000	0.0000	0.000 ± 0.000	0.000 ± 0.000	0.000 ± 0.000
Enterococcus faecalis	**0.4742**	**0.9999**	**0.3108**	0.036 ± 0.040	0.087 ± 0.097	0.023 ± 0.026
Staphylococcus aureus	0.1199	**0.9641**	0.0639	0.059 ± 0.086	0.060 ± 0.084	0.059 ± 0.088

Species	RAmpSim (5kb)			Uniform baseline (5kb)		
	F1 Score	Recall	Precision	F1 Score	Recall	Precision
Listeria monocytogenes	0.5033	0.5013	0.5052	0.477 ± 0.027	0.467 ± 0.039	0.488 ± 0.019
Pseudomonas aeruginosa	**0.2576**	**0.5578**	**0.1675**	0.200 ± 0.017	0.379 ± 0.033	0.136 ± 0.012
Bacillus subtilis	**0.3575**	0.4528	**0.2953**	0.230 ± 0.024	0.412 ± 0.046	0.160 ± 0.016
Escherichia coli	**0.7731**	**0.8023**	**0.7459**	0.344 ± 0.022	0.479 ± 0.032	0.268 ± 0.018
Salmonella enterica	**0.7140**	**0.8541**	**0.6134**	0.267 ± 0.028	0.353 ± 0.038	0.215 ± 0.022
Lactobacillus fermentum	0.0162	0.0682	0.0092	0.008 ± 0.068	0.007 ± 0.064	0.009 ± 0.072
Enterococcus faecalis	**0.5064**	**0.9997**	**0.3391**	0.051 ± 0.028	0.246 ± 0.133	0.028 ± 0.016
Staphylococcus aureus	0.0805	**0.9702**	0.0420	0.070 ± 0.108	0.067 ± 0.105	0.075 ± 0.111

Table S3. F1, recall, and precision scores of high/low coverage peak classification by RAmpSim and a uniform baseline classifier in the 2kb replicate (top) and 5kb replicate (bottom). Uniform baseline values are reported as mean ± CI. Compared to the 8kb replicate (Table 3), a larger number of species in the 2kb and 5kb replicates have RAmpSim F1, recall, and precision values that are close to, or fall within, the confidence intervals of the uniform baseline. This includes *P. aeruginosa* and *B. subtilis*, which exhibit more prominent background-like coverage distributions in the observed data. Performance similarly degrades for extremely low-abundance and low-coverage species such as *S. aureus*, where RAmpSim primarily struggles with precision, and for untargeted species such as *L. fermentum*, which show very little enrichment signal above background.

Replicate	Dataset	Target sensitivity	Strict specificity	Off-target burden
2 kb	Empirical	0.868	0.907	0.093
2 kb	RAmpSim	0.992	0.910	0.090
2 kb	Uniform	0.969	0.062	0.938
5 kb	Empirical	0.817	0.909	0.091
5 kb	RAmpSim	0.992	0.885	0.115
5 kb	Uniform	0.977	0.052	0.948
8 kb	Empirical	0.846	0.892	0.108
8 kb	RAmpSim	0.995	0.849	0.151
8 kb	Uniform	0.998	0.022	0.978

Table S4. Strict exact probe-footprint analysis of intended versus unintended capture. Probe-targeted bases were defined as the exact union of reference positions overlapped by aligned probes, with no padding or flanking allowance. For each dataset and fragment-size condition, target sensitivity was computed as the fraction of targeted bases covered by at least one read, strict specificity as the fraction of non-targeted bases receiving zero reads, and off-target burden as $1 -$ specificity. Values shown are global micro-averages across all reference bases for the primary setting (MAPQ ≥ 0, coverage threshold $\tau = 1$) for the empirical data, RAmpSim, and the uniform baseline. Because this analysis uses the exact probe footprint only, it provides a conservative assessment of intended versus unintended capture for long reads.

Table S5. Comparison of Theoretical, Observed, and Simulated Values for the 2kb and 5kb Replicates.

Species	Theoretical	2kb		5kb	
		Obs	RAmpSim	Obs	RAmpSim
Listeria monocytogenes	89.100000	44.255939	17.737358	57.308454	31.895452
Pseudomonas aeruginosa	8.900000	22.881611	33.129358	13.044702	28.086749
Bacillus subtilis	0.890000	9.634952	3.345772	9.719870	2.936187
Saccharomyces cerevisiae	0.890000	0.085608	0.205342	0.098807	0.304543
Escherichia coli	0.089000	14.470449	19.634965	12.186773	15.969443
Salmonella enterica	0.089000	8.635885	20.150204	7.609556	16.146896
Lactobacillus fermentum	0.008900	0.000564	0.204294	0.000266	0.185552
Enterococcus faecalis	0.000890	0.024360	2.340124	0.023562	1.844750
Cryptococcus neoformans	0.000890	0.000000	0.000000	0.000000	0.000000
Staphylococcus aureus	0.000089	0.010067	3.048291	0.007745	2.444875

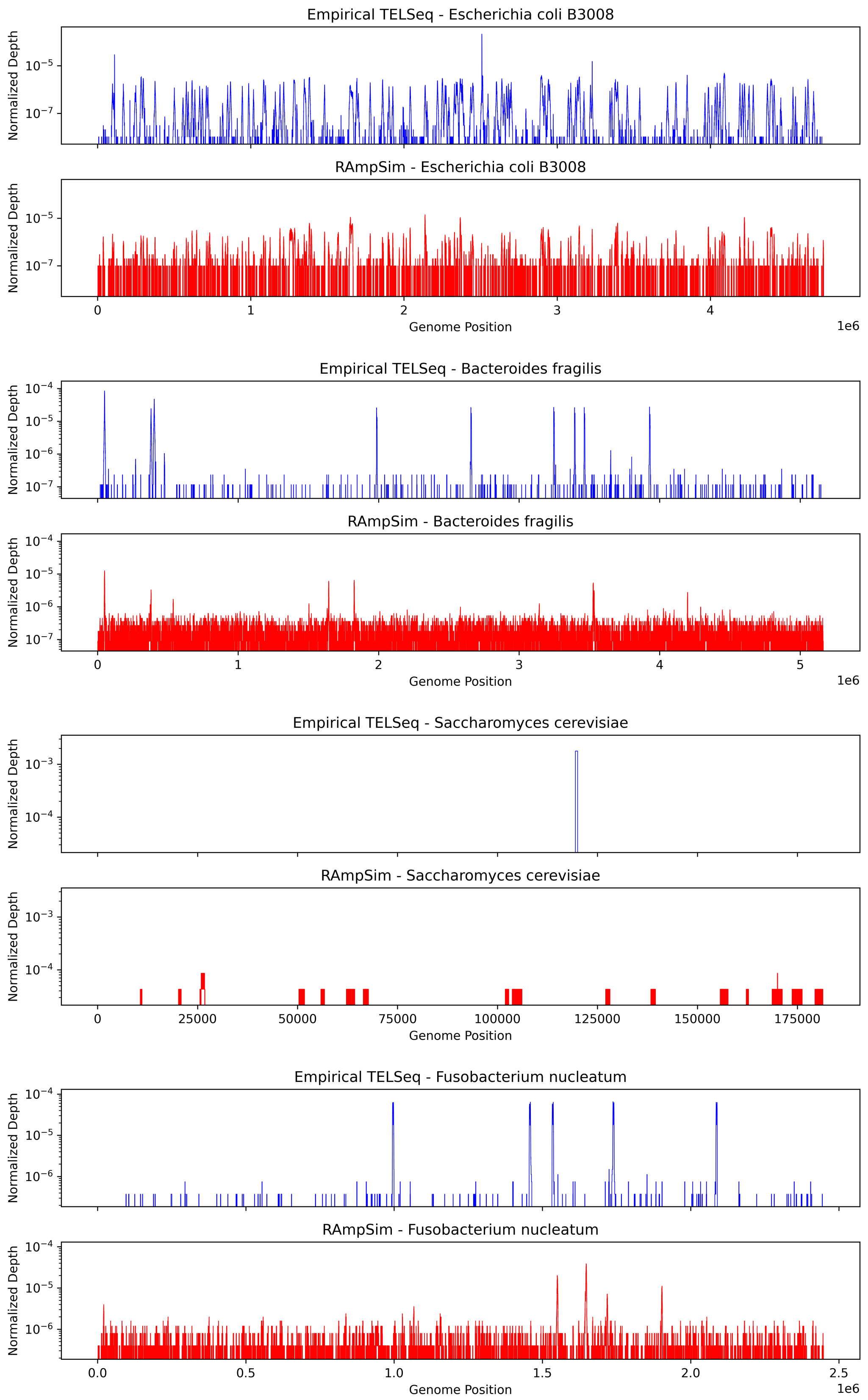

Fig. S6. Representative empirical-versus-simulated coverage profiles for the gut-mock supplementary analysis. Taxa were selected to illustrate a stronger-agreement case (*E. coli* B3008), a typical case (*B. fragilis*), a lower-agreement case (*S. cerevisiae*), and a GC-extreme/notable case (*F. nucleatum*).

Taxon	Case	EMD	F1
Escherichia coli B3008	Stronger agreement	0.053	0.530
Bacteroides fragilis	Typical case	0.143	0.145
Saccharomyces cerevisiae	Lower-agreement case	0.353	0.000
Fusobacterium nucleatum	GC-extreme/notable	0.132	0.103

Table S6. Representative taxa shown in the gut-mock supplementary coverage comparisons. As in the log distribution mock community, *S. cerevisiae* was not a target in the dataset.

**Species**	**Theoretical**	**Observed**	**Simulated**
Bacteroides fragilis	14.0000	1.3130	8.5759
Veillonella rogosae	14.0000	1.2308	4.1969
Faecalibacterium prausnitzii	14.0000	3.5210	5.7807
Roseburia hominis	14.0000	0.2756	5.5799
Fusobacterium nucleatum	6.0000	0.4083	1.9276
Bifidobacterium adolescentis	6.0000	0.2570	2.6121
Lactobacillus fermentum	6.0000	0.6104	2.1042
Prevotella corporis	6.0000	0.5091	2.0226
Escherichia coli B3008	2.8000	14.8263	7.6946
Escherichia coli B766	2.8000	17.5922	8.4239
Escherichia coli b2207	2.8000	14.3445	14.3115
Escherichia coli B1109	2.8000	15.4328	8.5817
Escherichia coli JM109	2.8000	22.5953	8.4258
Clostridioides difficile	1.5000	1.6021	2.1573
Akkermansia muciniphila	1.5000	0.0330	0.9454
Candida albicans	1.5000	0.00117	1.412478
Saccharomyces cerevisiae	1.4000	0.0008	1.8220
Methanobrevibacter smithii	0.1000	0.0038	0.0261
Salmonella enterica	0.0100	3.7048	11.1599
Enterococcus faecalis	0.0010	0.5747	3.2107
Clostridium perfringens	0.0001	1.1645	0.4411

Table S7. Species-level abundance comparison for the gut microbiome mock dataset. Abundance patterns relative to the theoretical distribution are somewhat similar between the observed and simulated data, with strongly targeted species (e.g. *E. coli*) increasing in abundance and several weakly targeted species (e.g. *B. fragilis*) decreasing in abundance.
